# Impact of Early Life Nutrition on Children’s Immune System and Noncommunicable Diseases Through Its Effects on the Bacterial Microbiome, Virome and Mycobiome

**DOI:** 10.3389/fimmu.2021.644269

**Published:** 2021-03-18

**Authors:** Paraskevi C. Fragkou, Dareilena Karaviti, Michael Zemlin, Chrysanthi Skevaki

**Affiliations:** ^1^ 4^th^ Department of Internal Medicine, Attikon University Hospital, National and Kapodistrian University of Athens, Athens, Greece; ^2^ 2^nd^ Department of Pediatrics, P. & A. Kyriakou Children’s Hospital, National and Kapodistrian University of Athens, Athens, Greece; ^3^ Neonatal Intensive Care Unit, Department of Pediatrics and Neonatology, Saarland University Medical Center, Homburg, Germany; ^4^ Institute of Laboratory Medicine, Universities of Giessen and Marburg Lung Center (UGMLC), Philipps University Marburg, German Center for Lung Research (DZL), Marburg, Germany

**Keywords:** microbiome, microbiota, virome, mycobiome, nutrition, children, immune system, noncommunicable diseases

## Abstract

The first 1000 days of life, including the intrauterine period, are regarded as a fundamental stepping stone for the development of a human. Unequivocally, nutrition during this period plays a key role on the proper development of a child, both directly through the intake of essential nutrients and indirectly by affecting the composition of the gut microbiota. The gut microbiota, including bacteria, viruses, fungi, protists and other microorganisms, is a highly modifiable and adaptive system that is influenced by diet, lifestyle, medicinal products and the environment. Reversely, it affects the immune system in multiple complex ways. Many noncommunicable diseases (NCDs) associated with dysbiosis are “programmed” during childhood. Nutrition is a potent determinant of the children’s microbiota composition and maturation and, therefore, a strong determinant of the NCDs’ programming. In this review we explore the interplay between nutrition during the first 1000 days of life, the gut microbiota, virome and mycobiome composition and the development of NCDs.

## Introduction

Nutrition early in life, including the intrauterine stage when the fetus is exposed to nutrients through maternal diet, plays a pivotal role not only on the growth of children but also on mental development and the initiation of numerous noncommunicable diseases (NCDs), like cardiovascular diseases, cancers, respiratory diseases, diabetes, atopy and allergies (**Supplementary Table 1**), that are possibly programmed during this period (1, 2). Childhood nutritional programming of adult diseases, the so called “fetal and infant origin of disease” hypothesis, underlines the interrelation between malnutrition during early life and the susceptibility to the development of various diseases during adulthood (3, 4). Therefore, nutrition acts as an important environmental factor that accelerates or ameliorates the genetically programmed predisposition into several NCDs (5).

Even though the importance of nutrition during the pre- and postnatal period of life on the ontogenesis of healthy children and, consequently, of healthy adults, has been well understood, the pathogenetic mechanisms beyond this phenomenon are far more complex than just the consumption of essential nutrients for cell and tissue growth. An increasing body of data supports that early-life nutrition is a potent determinant of the children gut microbiome assembly and maturation (6, 7). The gut microbiota, including bacteria, viruses, fungi, protists and other microorganisms (**Supplementary Table 1**), is a highly modifiable and adaptive system (7, 8). Its formation starts early in life, during the intrauterine development (9). The gut microbiota can be influenced by multiple factors such as the type of delivery (8, 10), maternal diet and body mass index, as well as the maternal health status prior to conception, while other parameters like the geographical region, domestic environment and feeding modes are also significant contributors  (2, 7, 11, 12). The interplay between postnatal nutrition, gut microbiota composition and abundance as well as their effects on children’s immune system and the development of NCDs is still poorly understood.

In this review we aim to present the effects of nutrition during the first 1000 days of life, including the stages of pregnancy, infancy and toddlerhood, on the children’s gut bacterial microbiota, virome and mycobiome, and the impact of these effects on the immune system and on the development of NCDs.

## Effects of Maternal Nutrition During Pregnancy on the Infant’s Bacterial Microbiota

The impact of a “balanced” healthy maternal nutrition during pregnancy on fueling an efficient fetal development and organogenesis has long been recognized. However, the exact effects of maternal nutrition on the formation of fetal microbiota during intrauterine life are still being investigated (13). Although evidence is still limited, increasing data by studies exploring the interplay between fetal exposure to an array of different nutrients though mother’s dietary habits, and the offspring’s bacterial microbiota composition, support that a correlation actually exists (**Table 1**) (11, 14). Indeed, a study examining both human subjects and non-human primates in mother – offspring dyads, found that meconium microbiota composition altered according to maternal diet; in particular, a high-fat, rich in refined carbohydrates (like sugar and processed grains) and animal products and low in greens and vegetables diet, was associated with remarkable alterations in the offspring’s intestinal bacterial community a notable relative depletion of *Bacteroides* in the meconium exposed to a high-fat diet during gestation (14). These changes were evident for up to 6 weeks after birth in neonates born by mothers who followed a high-fat type of diet (14). A study aiming to delineate the effects of maternal artificially sweetened beverages (ASB) consumption during pregnancy on the infants’ body mass index (BMI) and on their gut microbiome, showed that ASB-exposed infants had a divergent microbiome maturation trajectory, with community-level shifts in the bacterial taxa and depletion of several *Bacteroides* spp (20). Additionally, ASB-exposed infants had higher BMI at the age of one-year-old, than their non-exposed peers (20). Another study attempted to evaluate the influence of prenatal maternal diet on infants’ microbiota by dividing the cases according to mode of delivery. Results showed three main microbial clusters: *Bifidobacterium*, *Streptococcus* and *Clostridium*, *Bacteroides* and *Enterobacteriaceae* in infants born vaginally. Intriguingly, high fruit consumption by mothers during pregnancy was associated with a higher presence of *Clostridiaceae* and a lower presence of *Bifidobacterium* in vaginally born children (6). On the other hand, a greater concentration of the *Bifidobacterium* spp. was detected in babies delivered with cesarean section by mothers who followed a diet rich in red processed meat (6). Additionally, in the cesarean section subgroup, increased dairy intake favored the colonies of *Clostridium*, while seafood and fish ingestion enabled the colonization of the gut with group B *Streptococcus* in both vaginally and non-vaginally delivered neonates (6). The KOALA Birth Cohort study, investigated the association between vitamin D exposure during intrauterine life and the prevalence or abundance of specific bacterial taxa in the infants’ gut microbiota; the researchers found a significant negative linear trend between the dose of maternal vitamin D supplementation and the counts of *Bifidobacterium* spp. and *Clostridium* *difficile*, underlining that the levels of vitamin D during pregnancy affect the offspring’s gut microbiota composition (18). In a subset of 1,157 mother-infant pairs of the CHILD Cohort study, maternal consumption of vitamin-D fortified milk was associated with reduced the likelihood of *C. difficile* colonization in infants (17).

**Table 1:** Factors of maternal nutrition during pregnancy and associated modifications on the offspring’s microbiome.

Factors of nutrition during pregnancy	Effects on offspring’s microbiota
***Nutrition, vitamins and micronutrients supplementation during pregnancy***	
Maternal high fat diet (fat concentration >43.1%) in humans (14)	Maternal high fat diet associated with decreased numbers of *Bacteroidetes* colonies in neonates for up to 6 weeks postnatally
Maternal high fat diet in human and animal models (15)	Diminish of *Lactobacillus reuteri* in bacterial colonization of neonatal gut
Prenatal and postnatal maternal supplementation with iron and folic acid and multiple micronutrients (16)	No statistical significant alteration in α and β^*^ diversity of gut microbiome
Maternal consumption of lipid based nutrient supplements (prenatally and postnatally) (16)	Increased infant gut microbiota diversity at 18 months postpartum. No difference was noted regarding β diversity.
Prenatal maternal vitamin D supplementation (17)	No association with infant *Clostridium difficile* colonization in crude or adjusted models
Prenatal maternal milk consumption (>3 cups per day compared to less or 1 cup per day) (17)	Statistical significant lower odds of *Clostridium difficile* colonization in exclusively breastfed infants
Maternal vitamin D supplementation (18)	Infant’s gut microbiota is altered according to the dose of vitamin D supplementation during pregnancy
Maternal consumption of vitamin D fortified milk (17)	Reduced chances of *Clostridium difficile* colonization in infants
Maternal high fruit consumption among vaginally born infants (6)	Increased odds for high *Streptococcus/Clostridium* gut colonization
Maternal diet rich in red processed meat among babies delivered by caesarian section (6)	Increased numbers of *Bifidobacterium* in offspring.
Prenatal maternal consumption of non-nutrient sweeteners (19)	May alter maternal microbiome and therefore microbiome transmitted to offspring after birth. Association with obesogenic effects on offspring is possible.
Prenatal maternal consumption of artificially sweetened beverages (20)	Divergent microbiome maturation trajectory, with community-level shifts in the bacterial taxa and depletion of several *Bacteroides* spp. Association with obesogenic effects on offspring.
***Prebiotics and probiotics consumption during pregnancy***	
Supplementation with indigestible oligosaccharide prebiotics (fructo-oligosaccharides and galactooligosaccharides) (21)	Significant increase of the number of maternal fecal *Bifidobacterium* spp. especially *Bifidobacterium longum*. Bifidogenic effect not proved in neonates.
Probiotics consumption (22)	Alterations in vaginal microbiota and therefore the formation of neonatal gut microbiota might be influenced

^*^α diversity, Variation of microbes in a single sample; β diversity, Variation of microbial communities between samples.

## Effects of Breastfeeding on Neonates’ Bacterial Microbiota

Breastfeeding is widely accepted as the nutritional gold standard for infants. The protective role of breastmilk against numerous diseases such as diabetes and obesity as well as its benefits on the intellectual development of the children has been well established (23, 24). Breastmilk is rich not only in proteins (immunoglobulins, cytokines etc.), lipids (free fatty acids, phospholipids etc.) and the human milk oligosaccharides (HMOs) but also in bacteria, viruses and fungi (9, 25–29).

**Figure 1:**
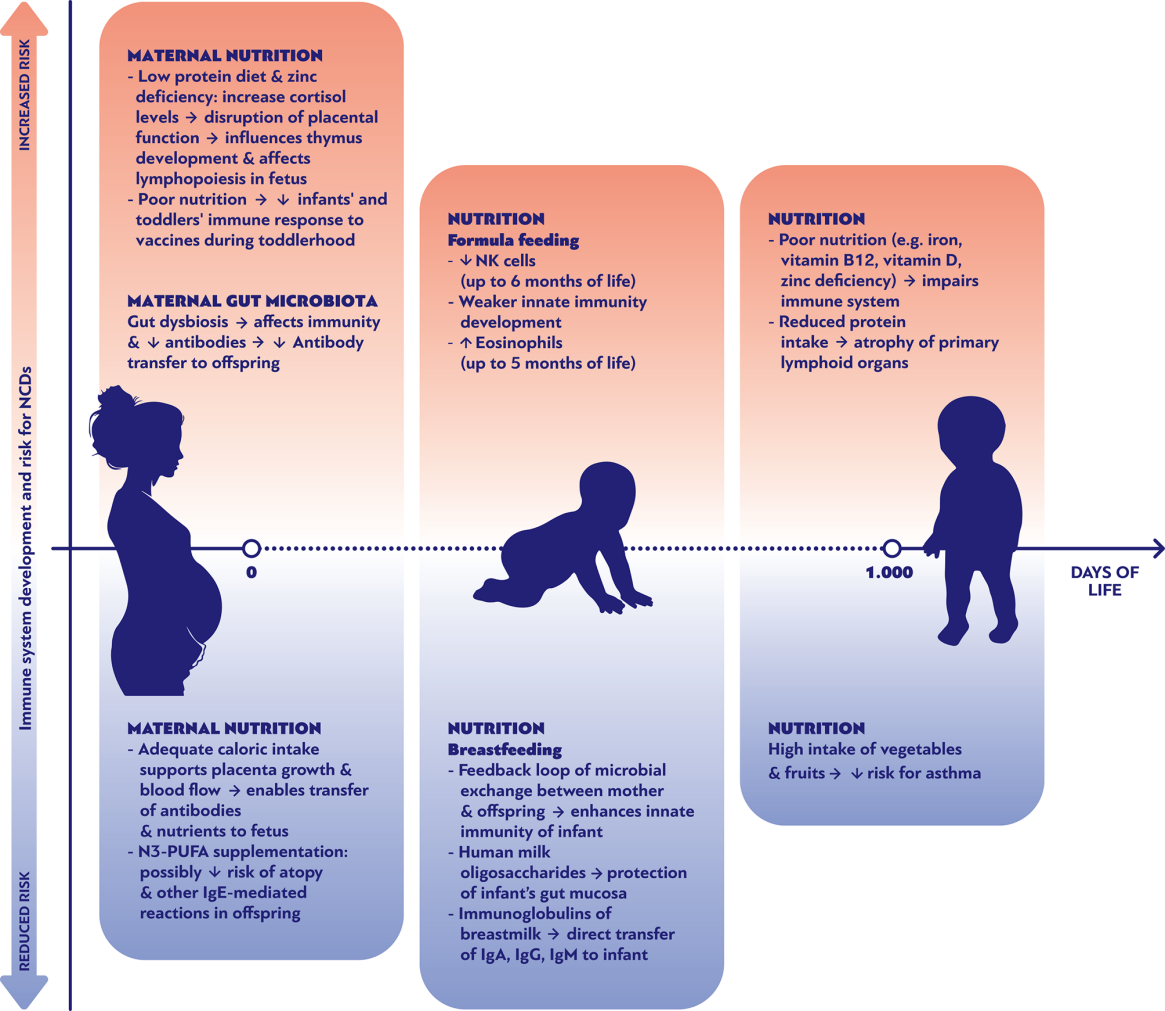
Nutritional parameters affecting positively or negatively the development of the immune system and the risk of noncommunicable diseases during the first 1000 days of life.

The breastmilk’s endogenous content in bacterial species functions as the foundation for the formation of a rich gut microbiota, providing essential nutrients for its growth and expansion (9, 30, 31). Interestingly, certain bacterial strains (including *Streptococcus* spp. and *Veillonella dispar*) co-occur both in mothers’ milk and in their offspring’ stool, a phenomenon that is diminished in infants fed with pumped breastmilk (32). Undoubtedly, the infant’s gut microbiota is significantly influenced by the “breastfeeding exclusivity and duration” (32). Moreover, feeding of infants with pumped breastmilk has been associated with enrichment of the breastmilk microbiota with potential pathogens as well as depletion of bifidobacteria, a phenomenon that highlights the hypothesis of retrograde microbial inoculation of the milk by the infants’ oral cavity (30). Conversely, studies have shown evidence of a feedback relation between the maternal skin and milk on one side and the baby’s’ saliva on the other. This feedback loop manifests itself as an exchange of microbiota and pathogens from mother to offspring and vice versa. It is believed that this mechanism structures a biochemical pathway that assists innate immunity in the neonatal period (**Figure 1**). Hence, the composition of the breastmilk is adjusted to the needs of the newborn by changing over time in order to modulate the formation of the gut microbiota. For instance, colostrum has high concentrations of HMOs, while mature milk contains greater amounts of protein. HMOs are not only the main nutrient for the saccharolytic gut microbiota species, but are also crucial for the development of the *Bifidocterium* spp. colonies that dominate the gut of healthy breastfed infants (8, 9, 31). On the contrary, formula-fed infants show a greater alpha-diversity in gastrointestinal bacterial colonies and decreased numbers of *Bifidobacterium* spp. and higher numbers of *Veillonella* and *Clostridioides* (26, 31, 33, 34). A recent prospective observational study in preterm infants in a neonatal Intensive Care Unit, which attempted to reveal the effects of breastmilk in the gut microbiota, showed that preterm babies fed either with their mother’s own milk (MOM) or with pasteurized donor’s human milk (DHM) had closer microbiota profiles compared to the group of formula fed infants. On the other hand, MOM-fed neonates had a significantly greater presence of *Bifidobacteriaceae* and lower presence of *Staphylococcaceae*, *Clostridiaceae*, and *Pasteurellaceae* compared to preterm DHM-fed infants (35). A small cohort with 10 formula-fed and 10 MOM-fed preterm infants, found a similar alpha-diversity but a significantly different beta-diversity of the gut microbiota between the two groups; additionally, the *Propionibacterium*, *Streptococcus*, and *Finegoldia* genera and bacteria of the *Clostridiales* order had significantly higher relative abundance in the MOM group, while bacteria of the *Enterobacteriaceae* family, the *Enterococcus* and *Veillonella* genera, and *Bacilli* class were more abundant in the formula group (36).

## Effects of Introduction of Infant Formulas and Solid Food on Children’s Bacterial Microbiota

Although many studies have explored the short and long-term influence of the mode of birth and the type of milk (breastmilk versus formula) on the infants’ flora composition (33, 37–40), fewer studies have addressed the second phase of nutrition, which could be defined as the time after the introduction of solid food and prior to weaning, and the third phase of nutrition that begins with weaning.

The introduction of solid food leads to a significant diversification of nutrients from the relative homogeneous components of the breast or bovine milk towards plant and meat-derived meals, including the microbial content of these foods. Moreover, it coincides with the “oral stage” of development, which is associated with the uptake of a great variety of microbes through insertion of environmental objects into the mouth. Thus, the maturation of the “enterotypes” reflects a mixture of influences by food and multiple environmental exposures (41). Interestingly, this diversification of microbial exposure is associated with an inter-individual convergence of the microbiota, thus neonatal microbiota differ more from each other than the more mature microbiota of infants and children (42, 43). The introduction of solid foods is associated with a decrease in *Bifodobacterium* and an increase in Firmicutes phylum (42, 44). The introduction of soy formula milk was associated with an increase in *Lachnospiraceae*. This results in the enhancement of metabolic pathways suggesting a short-chain fatty acid (SCFA)-rich environment, including glycerol to 1-butanol fermentation which has been associated with dysbiosis (43). Intriguingly, *Bifidobacteriaceae* were reduced in infants prior to the introduction of soy formula. The introduction of soy formula could potentially reflect a reaction to gastrointestinal symptoms that can be interpreted as an intolerance to milk-based nutrition (43). This might be an early example for a microbiome-driven modification of nutritional habits which also may play a great role during later life. Moreover, the composition of the infant’s microbiome correlates with the domestic water source (tap water versus boiled/distilled water), probably due to differing microbial or chemical components (43).

## Effects of Nutrition on Children Virome and Mycobiome

The virome of the human gastrointestinal tract consists mainly of eukaryotic viruses and bacteriophages (45). Bacteriophages are believed to play a fundamental role in enhancing the balance of microbiota and are probably responsible for the microbiome – virome interaction (46). The colonization of children’s intestine with viral species as well as its association with maternal diet has not been thoroughly studied so far and, therefore, a correlation between maternal nutritional profile and virome formation in neonates is yet to be established. A detailed analysis of fecal DNA virome from Malawian twins along with maternal stool samples suggested that age is the most determining factor that defines the composition of the gut virome (46). The same result was confirmed in a meta-analysis of individuals of Western background, whereby intestinal virome was found to be mainly dependent on age, even in members of the same family who shared the same external environment (47). The greatest viral diversity has been noticed in infants aged 0-3 years and in adults aged 18-65 years (48).

The postnatal diversification of the eucaryotic virome appears to be triggered by environmental exposure, in particular by the exposure to breastmilk or formula milk (47, 49). Recent studies have attempted to investigate the interrelation between breastfeeding and gut virome composition in neonates (29, 47, 49). In general, the virome and especially the presence of bacteriophages, is crucial for a balanced intestinal microenvironment (50). Intriguingly, the bacteriophage virome appears to be guided by the initial bacterial pioneer strains after birth. Moreover, an interesting study indicated that bacteriophages from milk are transferred to the neonate’s gut during breastfeeding (29). Additionally, the protective role of breastmilk against multiple viruses is a common ground, as supported by data showing an abundance of human viruses in fecal samples from formula-fed neonates compared to a weaker viral gut colonization in breastfed babies (49). The bacteriophage virome contracts during the first 2 years of life due to an increasing predominance of microviridae that reach 80% of the relative abundance at 24 months of age (47).

The human mycobiome consists of fewer strains than the bacterial microbiome (51). Preterm birth is associated with a predominance of saccharomyces, specifically *Candida*, in the meconium (52). Thus, the mycobiome may diversify during ontogeny paralleling the bacterial microbiome. However later in life, the human mycobiome is characterized by a relatively stable core mycobiome which is dominated by yeasts. A prospective study examining the mycobiota in mother-offspring dyads detected fungal colonies in the gut of newborns around the age of 2 weeks (53). The same study demonstrated traces of fungal DNA in the majority of both members of the same dyad, while maternal consumption of probiotics during pregnancy was associated with an increased concentration of maternal mycobiota (53). Another study reported that pre-, pro- and antibiotics dietary consumption have an effect on mycobiome formation in the infant’s oral cavity and gastrointestinal tract (54). Breastmilk is believed to be a transmitter of fungi, as studies have revealed the presence of *Malassezia*, *Candida* and *Saccharomyces* species in milk samples. A study examining the milk mycobiota from 271 mothers from the CHILD birth cohort, revealed that fungi were isolated in 21.4% of the mothers; the most dominant species were *Candida*, *Alternaria*, and *Rhodotorula* (25). Interestingly, the milk samples where fungi were isolated from, had lower concentrations of two HMOs (25). Data on the possible translocations of fungal species during breastfeeding between maternal skin and the infant’s oral cavity are scarce (55, 56). The transfer of fungal species from mother to infant and its significance to their health is increasingly being examined, although-unlike adult gut mycobiota data-the evidence is still limited. However, in a longitudinal study, the interindividual variations were as pronounced as the intraindividual variations over time (51). This may indicate that the mycobiome depends on environmental factors that are yet to be determined.

## Early Life Microbiota Effects on Immunity and the Development of NCDs

The interaction between early life microbiota and the development of the immune system is quite complex. The exposure to microorganisms (either pathogenic or innocent bystanders, like normal flora) serves as an “immune-educator” that sets the foundations for sustainable immune responses which distinguish between self, non-self and pathogenic antigens (57). Nonpathogenic microbes evoke an immunosuppressive effect on intestinal epithelial cells by inhibiting the transcription factor nuclear factor (NF)-κB pathway, thus demonstrating a direct anti-inflammatory effect (58, 59). The key-role of “immune-education” is supported by data showing that probiotic bacteria may be protective against atopic disease by blunting a T helper (Th)2-skewed immune response (57). Moreover, the institution of antigen-tolerance bath in gut and systematically, can also be dependent on suppressive cytokines, such as IL-10 and transforming growth factor (TGF)-β produced by regulatory T cells, that control the activity of other cells like the Th1 and Th2 (58). The effect of gut microbial colonization on the development of the immune system, and its potential impact on programming of NCDs (such as atopy), was also supported by a study in newborns depicting that putative CD4+CD25+ regulatory T cells were expanded in infants colonized by toxin-producing *Staphylococcus aureus* early in life, compared to non-colonized infants or those who had a later colonization; children who developed allergy within the next 18 months, were significantly less often colonized by toxin-producing *S. aureus* during the first days after birth (57).

The link between the influences of inflammatory and metabolic pathways induced by different nutritionally-driven microbiome profiles and the determination of NCDs development has not been concretely established yet. NCDs are unequivocally multifactorial, and microbiome effects during childhood on their development later in life are only one piece of the puzzle, among others like genetic predisposition, environmental factors and lifestyle habits. The complexity of this puzzle is immense since it involves the interactions between the bacterial microbiome, virome, and mycobiome on one side, and virtually all organ systems of the host on the other (40). Notwithstanding, the advent of transcriptomics, microbial genomics and metabolomics has substantially contributed in deciphering the role of microbiome in modulating the immune system towards or away from the development of NCDs (60). For instance, animal studies have shown that gut microbiota of high fiber fed animals produce metabolites (short chain fatty acids, SCFAs) that induce T-regulatory lymphocyte populations and modulate bone marrow-derived antigen-presenting cell precursors, and these in turn, reduce dendritic cell proliferation and Th2 reactivation in the lungs, thus providing a possible protective mechanism against asthma development (60).

Understanding the link between early antigen exposures and programming of NCDs is a prerequisite for new therapeutic strategies. Specific diet recommendations might contribute in reducing the chances to develop specific types of NCDs. As shown in **Figure 1**, multiple nutritional factors can either promote or ameliorate the risk of NCDs through their effects on immune system development during pregnancy, early and late infancy period. For example, the maternal consumption of omega-3 fatty acid supplements during pregnancy has been associated with decreased risk of food allergies and other IgE mediated conditions during the first 12 months of life (**Figure 1**) (61). Genetic and epigenetic predispositions lead to a myriad of individual fates, indicating that there cannot be a “one size fits all” optimal microbiome, virome and mycobiome to prevent NCDs. However, large scale “omics” research has revealed distinct colonization patterns with prognostic relevance (40, 47, 62, 63). In general, it appears that shifts within the colonization often affect the risk of various NCDs in the same direction. Thus, it does not seem as if the implementation of preventive interventions against the development of one group of NCDs (such as allergies) would lead to an increased risk for another group of NCDs (such as obesity).

The central hypothesis may be that vaginal delivery, (long term) breastfeeding, early exposure to a wide variety of foods including potential allergens and a farm environment, as well as the avoidance of antibiotics and tobacco smoke are associated with “eubiosis” i.e. “a balanced host-microbe interaction (‘healthy’ microbiome)” (**Supplementary Table 1**) and a reduced risk of NCDs (9, 40, 64). Regarding the microbiome, “eubiosis” is characterized by a predominance of *Bifidobacterium*, *Lactobacillus* and *Veillonella* species during the time of exclusive breast feeding, whereas *Bacteroides* and *Clostridiales* dominate after the introduction of solid foods (40, 44). The gut microbiome of children born by caesarean section who are more prone to develop allergies later in life, is dominated by *Enterobacteriaceae* (38). The neonatal microbiome of infants that are born by caesarean section can be modified to resemble the microbiome after vaginal birth by postnatal inoculation with vaginal swabs from the mother (10). Although the long term effects are still under investigation, this study is regarded as a proof of principle that the neonatal microbiome can be modified successfully.

Little is known about the influence of the neonatal virome on the risk of NCDs. The virome may play multiple roles by either colonizing or infecting the host or by modifying the microbiome in the form of bacteriophages. The gut mycobiome regulates the host immunity and affects the course of chronic inflammatory diseases (65). However, it is still unclear whether the mycobiome has direct or indirect influences on the infant’s risk of developing NCDs.

## Research Gaps and Future Research Needs

Although increasing evidence supports the association between pre- and postnatal nutrition of children and modifications of their gut microbial community, the interplay between nutrition and microbiome composition in other body sites remains obscure. Moreover, data regarding the effects of certain types of diet (like the vegetarian, the vegan, the ketogenic etc.) on the gut microbiome during gestation, during the phase of solid food introduction, as well as later in childhood are limited. Also, little is still known about the complex interactions between the specific modifications of microbiome during this period of life and its impact on immunobiological mechanisms that are associated with the development of NCDs later in life. Intriguingly, based on recent evidence that bacterial strains, once acquired, can be retained in gut microenvironment for a long time (66), research efforts should be made on exploring the reversibility of the microbiome-induced immune effects over time in children with nutritionally-induced dysbiosis. Finally, more data are needed regarding the nutritionally-driven alterations of virome and mycobiome during early life and how these are associated with morbidity phenotypes later in life.

## Conclusions

Nutrition during the first 1000 days of life is unequivocally important for a prosperous developmental trajectory of children and for a healthier “programming” of adult life. Nutrition has a multidimensional impact on ontogenesis, including the modification of our microbiome, which in turn interacts with the immune system and promotes or halts numerous NCDs. The knowledge gained thus far, from adult and, to a lesser extent, from pediatric studies should guide further research and catalyze an attitude towards healthier and more balanced nutritional approaches, especially during the first years of life.

## Author Contributions

CS and PF planned, structured and edited the manuscript. PF searched the literature and integrated all contributions. PF, DK and MZ participated in the writing of first draft of manuscript and subsequent revisions. All authors contributed to the article and approved the submitted version.

## Funding

CS is supported by the Universities Giessen and Marburg Lung Center (UGMLC), the German Center for Lung Research (DZL), University Hospital Giessen and Marburg (UKGM) research funding according to article 2, section 3 cooperation agreement, and the Deutsche Forschungsgemeinschaft (DFG)-funded SFB 1021 (C04), KFO 309 (P10), and SK 317/1-1 (Project number 428518790) as well as by the Foundation for Pathobiochemistry and Molecular Diagnostics. PF is supported by Doctorate scholarship by the State Scholarships Foundation (IKY), Partnership Agreement (PA) 2014-2020, co-financed by Greece and the European Union (European Social Fund - ESF) through the Operational Programme “Human Resources Development, Education and Lifelong Learning 2014-2020”.

## Conflict of Interest

For CS: Consultancy and research funding, Hycor Biomedical, BencardAllergie and Thermo Fisher Scientific; Research Funding, Mead Johnson Nutrition (MJN).

The remaining authors declare that the research was conducted in the absence of any commercial or financial relationships that could be construed as a potential conflict of interest.
