# Supplementary material for: Impact of Early Life Nutrition on Children’s Immune System and Noncommunicable Diseases Through Its Effects on the Bacterial Microbiome, Virome and Mycobiome
Source: Front Immunol. 2021 Mar 18;12:644269. doi: 10.3389/fimmu.2021.644269 (PMC8012492; doi:10.3389/fimmu.2021.644269)
Supplement: Supplementary file 1 [file Table_1.docx]

Supplementary Material

**Supplementary Table 1**. Definitions of terms (glossary) used in this review.

| **Terms** | **Definitions** | **Source / Reference** |
| --- | --- | --- |
| Dysbiosis | “Changes in quantitative and qualitative composition of microbiota. The changes may lead to altered host microbial interaction or homeostatic imbalance that can contribute to a disease state often with inflammation.” | Adopted from: the Medical Subject Headings (MeSH) terms (available from: <https://www.ncbi.nlm.nih.gov/mesh>, accessed on 19 Feb 2021). |
| Eubiosis | “Refers to a balanced host-microbe interaction (“healthy” microbiome).” | Adopted from: Berg G, Rybakova D, Fischer D, Cernava T, Champomier Vergès MC, Charles T, Chen X, et al. *Microbiome* definition re-visited: old concepts and new challenges. Microbiome (2020) **8**: 103. doi: 10.1186/s40168-020-00875-0. |
| Microbiome | “Contains both the microbiota (community of microorganisms) and their “theatre of activity” (structural elements, metabolites/signal molecules, and the surrounding environmental conditions).” | Adopted from: Berg G, Rybakova D, Fischer D, Cernava T, Champomier Vergès MC, Charles T, Chen X, et al. *Microbiome* definition re-visited: old concepts and new challenges. Microbiome (2020) **8**: 103. doi: 10.1186/s40168-020-00875-0. |
| Microbiota | “The full collection of microbes (bacteria, fungi, virus, etc.) that naturally exist within a particular biological niche such as an organism, soil, a body of water, etc.” | Adopted from: the Medical Subject Headings (MeSH) terms (available from: <https://www.ncbi.nlm.nih.gov/mesh>, accessed on 19 Feb 2021). |
| Mycobiome | “The full spectrum of fungi that exist within a particular biological niche such as an organism, soil, a body of water, etc.” | Adopted from: the Medical Subject Headings (MeSH) terms (available from: <https://www.ncbi.nlm.nih.gov/mesh>, accessed on 19 Feb 2021). |
| Noncommunicable diseases | “Diseases which are typically non-infectious in origin and do not transmit from an affected individual to others. The four main types of noncommunicable diseases are cardiovascular diseases (e.g., heart attacks and stroke), cancer, chronic respiratory diseases (e.g., chronic obstructive pulmonary disease and asthma) and diabetes mellitus.” | Adopted from: the Medical Subject Headings (MeSH) terms (available from: <https://www.ncbi.nlm.nih.gov/mesh>, accessed on 19 Feb 2021). |
| Virome | “All of the viruses that exist within a particular biological niche such as an organism or specific location of the organism, soil, a body of water, etc.” | Adopted from: the Medical Subject Headings (MeSH) terms (available from: <https://www.ncbi.nlm.nih.gov/mesh>, accessed on 19 Feb 2021). |
